# Rethinking medulloblastoma from a targeted therapeutics perspective

**DOI:** 10.1007/s11060-018-2917-2

**Published:** 2018-06-05

**Authors:** Yuuri Hashimoto, Marta Penas-Prado, Shouhao Zhou, Jun Wei, Soumen Khatua, Tiffany R. Hodges, Nader Sanai, Joanne Xiu, Zoran Gatalica, Lyndon Kim, Santosh Kesari, Ganesh Rao, David Spetzler, Amy Heimberger

**Affiliations:** 1 Department of Neurosurgery, The University of Texas M.D. Anderson Cancer Center, Houston, TX USA; 2 Department of Neuro-Oncology, The University of Texas M.D. Anderson Cancer Center, Houston, TX USA; 3 Department of Biostatistics, The University of Texas M.D. Anderson Cancer Center, Houston, TX USA; 4 Department of Pediatrics, The University of Texas M.D. Anderson Cancer Center, Houston, TX USA; 5 Division of Neurosurgical Oncology, Barrow Neurological Institute, Phoenix, AZ USA; 6 Caris Life Sciences, Phoenix, AZ USA; 7 Department of Neurological Surgery and Medical Oncology, Thomas Jefferson University Hospital, Philadelphia, PA USA; 8 Department of Translational Neurosciences and Neurotherapeutics, Pacific Neuroscience Institute and John Wayne Cancer Institute at Providence Saint John’s Health Center, Santa Monica, CA USA; 9 Department of Neurosurgery, The University of Texas MD Anderson Cancer Center, Unit 442, Houston, TX 77030 USA

**Keywords:** Medulloblastoma, Molecular profiling, Targeted therapy

## Abstract

**Introduction:**

Medulloblastoma is an aggressive but potentially curable central nervous system tumor that remains a treatment challenge. Analysis of therapeutic targets can provide opportunities for the selection of agents.

**Methods:**

Using multiplatform analysis, 36 medulloblastomas were extensively profiled from 2009 to 2015. Immunohistochemistry, next generation sequencing, chromogenic in situ hybridization, and fluorescence in situ hybridization were used to identify overexpressed proteins, immune checkpoint expression, mutations, tumor mutational load, and gene amplifications.

**Results:**

High expression of MRP1 (89%, 8/9 tumors), TUBB3 (86%, 18/21 tumors), PTEN (85%, 28/33 tumors), TOP2A (84%, 26/31 tumors), thymidylate synthase (TS; 80%, 24/30 tumors), RRM1 (71%, 15/21 tumors), and TOP1 (63%, 19/30 tumors) were found in medulloblastoma. TOP1 was found to be enriched in metastatic tumors (90%; 9/10) relative to posterior fossa cases (50%; 10/20) (p = 0.0485, Fisher exact test), and there was a positive correlation between TOP2A and TOP1 expression (p = 0.0472). PD-1 + T cell tumor infiltration was rare, PD-L1 tumor expression was uncommon, and TML was low, indicating that immune checkpoint inhibitors as a monotherapy should not necessarily be prioritized for therapeutic consideration based on biomarker expression. Gene amplifications such as those of *Her2* or *EGFR* were not found. Several unique mutations were identified, but their rarity indicates large-scale screening efforts would be necessary to identify sufficient patients for clinical trial inclusion.

**Conclusions:**

Therapeutics are available for several of the frequently expressed targets, providing a justification for their consideration in the setting of medulloblastoma.

**Electronic supplementary material:**

The online version of this article (10.1007/s11060-018-2917-2) contains supplementary material, which is available to authorized users.

## Introduction

Medulloblastoma is the most common malignant central nervous system (CNS) pediatric tumor and also occurs in adults, albeit less frequently. Clinical prognosis and stratification are dependent on clinical variables such as age, presence of metastasis inside or outside the CNS, and extent of surgical resection [1]. Recently, in addition to histological classification, molecular subgroups (WNT, SHH, Group 3 and Group 4) with distinct clinical and genomic characteristics have been identified as important prognostic factors in larger retrospective series and are now being validated prospectively [2]. Current treatment paradigms are based on risk stratification (standard-risk and high-risk for recurrence) and involve multimodal therapeutic approaches (surgery, craniospinal radiation, chemotherapy). These treatment strategies have shown an improvement in 5-year overall survival to 85% for children with standard-risk disease and ~ 60% for those with high-risk disease [3]. However, long-term survival is often associated with treatment-related morbidity, and late relapses are still possible, particularly in adult medulloblastomas [4]. Targeted therapeutics with agents such as vismodegib and other smoothened (SMO) inhibitors are of potential benefit to only a single subgroup, the SHH-subtype that has sonic hedgehog pathway activation, constituting approximately 30% of medulloblastoma patients in children and more than 50% in adults [5, 6]. Because multiple alterations define these subsets, careful genomic and molecular classification is required to discover new actionable targets, particularly for groups 3 (the subtype with worse outcome) and 4 (the most frequent subtype in children and the second in adults), for which no targeted agents are yet available [7]. Therefore, we hypothesized that precision medicine profiling would be informative regarding applicable targeted therapeutic strategies and biomarker-based chemotherapies of potential benefit for medulloblastoma patients that pose a treatment quandary for the clinician.

## Materials and methods

We analyzed 36 medulloblastomas (18 pediatric and 18 adult samples) submitted to Caris Life Sciences for multiplatform analysis (e.g., sequencing, immunohistochemistry) (Supplementary Tables 1, 2 and 3). Prior treatment histories and clinical annotation are not provided by the referring physicians; however, one submitted pediatric case (1/18) and 44% of the adult cases (8/18) were designated as “recurrent” as part of their submitted diagnosis. IHC analysis was performed on sections on full slides from formalin-fixed paraffin-embedded (FFPE) tumor specimens. Abiding by the requirements of the Clinical Laboratory Improvement Amendments/Compliance Assistance Office (CLIA) and International Organization for Standardization, staining conditions were optimized and validated, and staining was performed per the manufacturer’s instructions using automated staining techniques. The results were evaluated and confirmed by independent board-certified pathologists. Results were categorized as positive or negative by defined thresholds specific to each marker [8], based on published clinical literature that associates biomarker status with patient responses to therapeutic agents.

Using the Illumina MiSeq and NextSeq platforms, next generation sequencing (NGS) was performed on genomic DNA. DNA was isolated from microdissected FFPE tissue using QIAamp DNA FFPE DNA Extraction Kit. Specific regions of either 47 or 592 genes (a panel of pan-cancer genes of interest related to cancer genomics based on current literature http://www.carismolecularintelligence.com/tumor-profiling-menu/) were amplified and enriched using the customized Illumina TruSeq Amplicon Cancer Hotspot panel and Agilent custom-designed SureSelect XT assay [9]. All variants reported were detected with > 99% confidence based on the mutation frequency present. Tumor mutational load was calculated by counting all non-synonymous missense mutations that had not previously been reported as germline alterations. The NextSEQ platform sequences a total of 592-cancer-related genes with a total sequenced length of 1.4 megabases. Even though whole exosome sequencing has been previously used to measure TML, in some cases smaller gene panels have been used and associated with immunotherapy response [10, 11]. Work previously published has also illustrated that interrogating mutations at the coding regions of a targeted gene panel can generate TML values that are highly correlated with whole exome sequencing [12]. While whole exosome sequencing is only performed in research settings, using targeted sequencing allows for evaluation of TML in clinical settings. Copy number variation (CNV) was tested by NGS and was determined by comparing the depth of sequencing of genomic loci to a diploid control as well as the known performance of these genomic loci. Calculated gains ≥ 6 copies were considered amplified.

Gene amplifications were assessed using FISH for *EGFR* [EGFR/CEP7 probe] and CISH for *Her2* [INFORM HER-2 Dual ISH DNA Probe Cocktail]. *EGFR* amplification was defined by the presence of an EGFR/CEP7 ratio of ≥ 2, or ≥ 15 *EGFR* copies per cell in ≥ 10% of analyzed cells. All reported *P* values were two sided and corrected for multiple comparison. *P* values of less than 0.05 were declared as statistically significant. All analyses were performed with statistical software R *v3.3.1*.

## Results

**Fig. 1:**
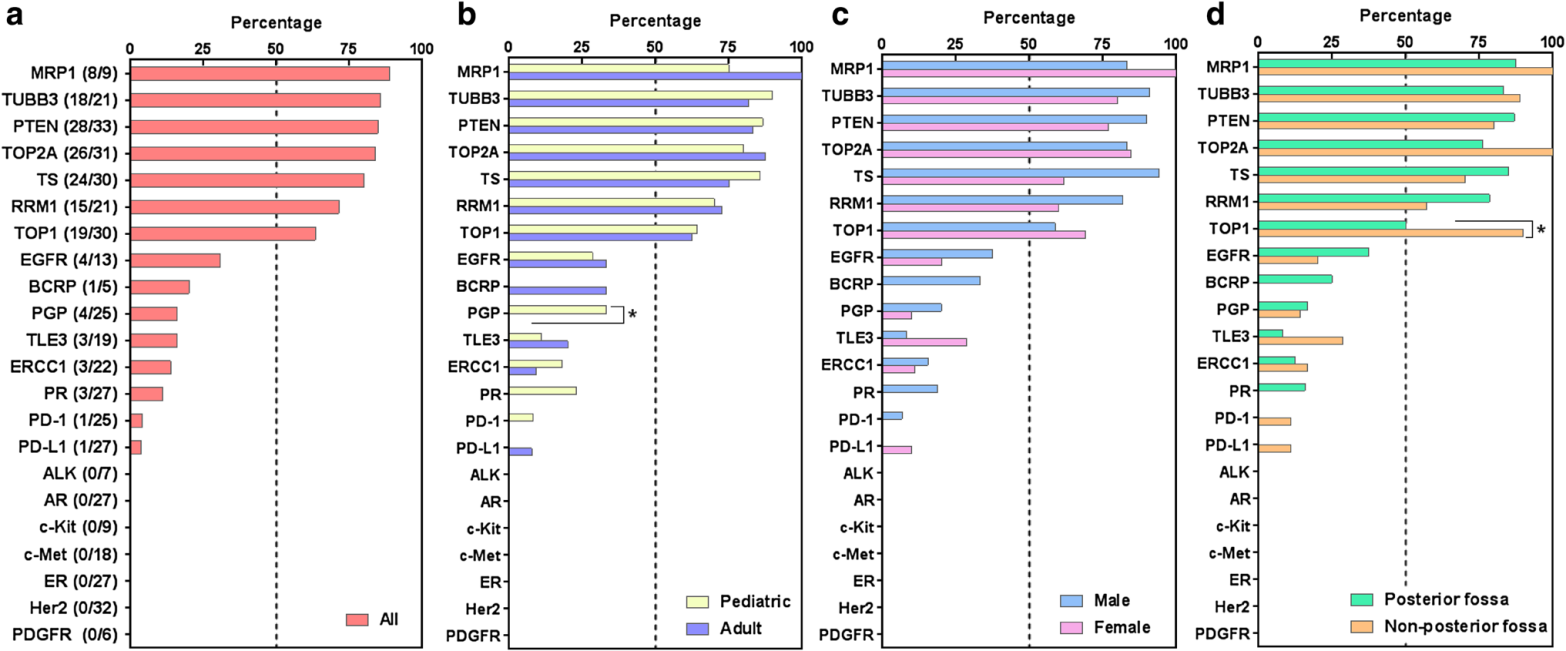
Percentage of medulloblastoma patients with designated protein expression. Expression frequency of all patients (**a**) and subgroups according to age (**b**), sex (**c**), and tumor location (**d**). *p < 0.05. Non-posterior fossa cases designate metastatic cases

**Fig. 2:**
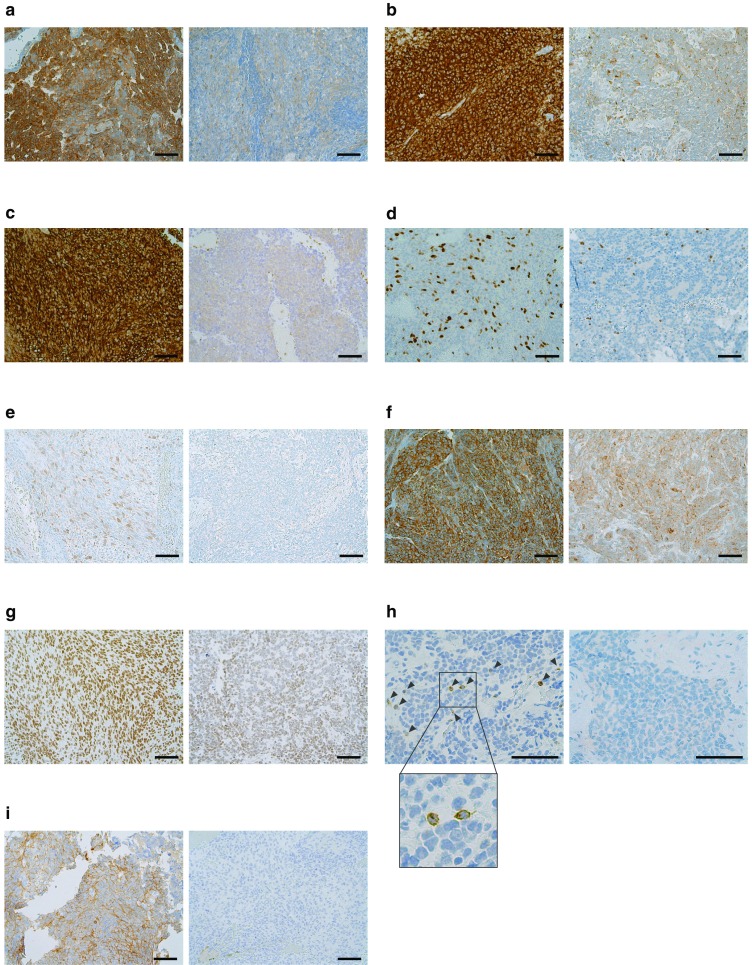
Representative immunohistochemical staining of MRP1 (**a**), TUBB3 (**b**), PTEN (**c**), TOP2A (**d**), thymidylate synthase (**e**), RRM1 (**f**), TOP1 (**g**), PD-1 on tumor infiltrating lymphocytes (**h**), and PD-L1 on tumor cells (**i**). A representative positive (left) and a negative (right) samples for each marker are shown. Bar 100 µm. Arrows show PD-1 positive T cells

Patient characteristics and the number of tumor specimens are shown in Table 1, which are consistent with known demographic features, including preferential enrichment in males in the pediatric population. This cohort contains tumors located outside the posterior fossa, which were recurrent or metastatic medulloblastomas. The most common cancer-associated biomarkers identified by IHC were the multidrug resistance-associated protein 1 (MRP1) (89%; 8/9 tumors), tubulin beta 3 class III (TUBB3) (86%; 18/21 tumors), phosphatase and tensin homolog (PTEN) (85%; 28/33 tumors), topoisomerase 2A (TOP2A) (84%; 26/31 tumors), thymidylate synthase (TS) (80%; 24/31 tumors); ribonucleotide reductase M1 (RRM1) (71%; 15/21 tumors), and topoisomerase 1 (TOP1) (63%; 19/30 tumors) (Fig. 1). These findings are consistent with mRNA levels in the data set of medulloblastoma patients from the TCGA although there are some anticipated differences secondary to post-transcriptional and epigenetic regulation (Supplementary Fig. 1). Representative IHC results are shown in Fig. 2. TOP1 was found to be enriched in metastatic tumors (90%; 9/10) relative to posterior fossa cases (50%; 10/20) (p = 0.0485, Fisher exact test) (Fig. 1), and there was a positive correlation between TOP2A and TOP1 expression (p = 0.0472). PD-1 + infiltrating T cells and tumor PD-L1 expression were low in medulloblastoma (Figs. 1, 2). PGP expression was only found in pediatric medulloblastoma cases (Fig. 1).

**Table 1:** Summary of characteristics of patients with medulloblastoma

	All	Pediatric	Adult
Number of patients	36	18	18
Age			
Mean, years (range)	19.6 (2–47)	7.7 (2–14)	31.6 (18–47)
Sex			
Male, *n* (%)	23 (63.9%)	14 (77.8%)	9 (50.0%)
Female, *n* (%)	13 (36.1%)	4 (22.2%)	9 (50.0%)
Tumor location			
Posterior fossa, *n* (%)	26 (72.2%)	15 (83.3%)	11 (61.1%)
Non-posterior fossa, *n* (%)	10 (27.8%)	3 (16.7%)	7 (38.9%)

**Fig. 3:**
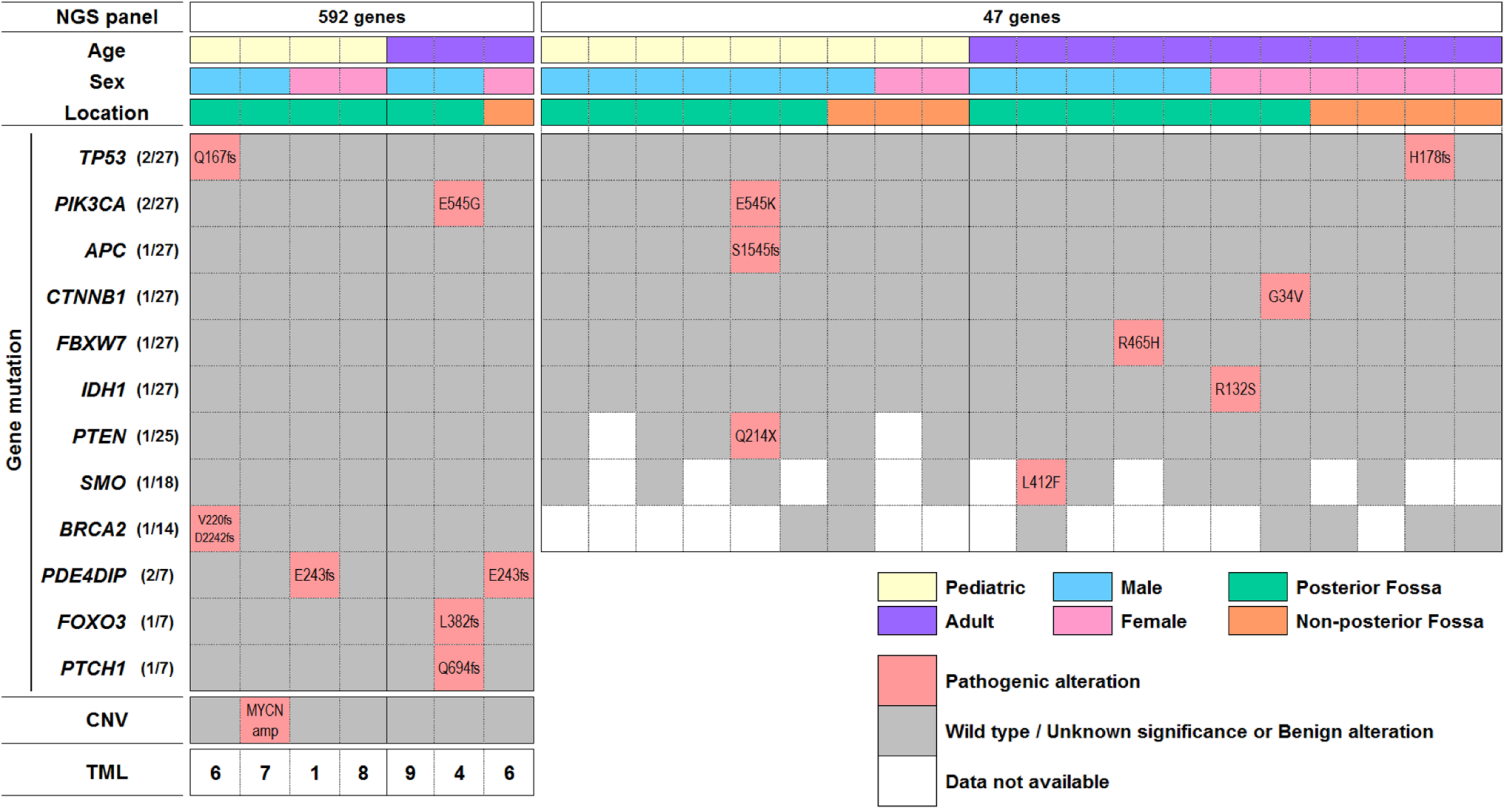
Gene mutation distribution in the medulloblastoma cohort. Gene mutations that have been reported as pathogenic for malignancies are shown. The variants were interpreted by board-certified molecular geneticists and categorized as pathogenic, presumed pathogenic, variant of unknown significance, presumed benign, or benign, according to American College of Medical Genetics and Genomics (ACMG) standards. Essentially a pathogenic variant has the highest confidence that it is disease causing or contributes to the disease, while benign has the lowest likelihood to cause disease. *CNV* copy number variant, *TML* tumor mutational load (per Mb). Non-posterior fossa cases designate metastatic cases

Mutational testing on individual samples was performed at the discretion of the ordering physician. Hence, not all samples were tested for all mutations. Nonetheless, among 27 tumors (13 pediatric, 14 adult) sequenced for either 47 or 592 genes, 2 had mutations in *TP53* (Q167fs, H178fs), *PIK3CA* (E545G, E546K), and *PDE4DIP* (E243fs), and one mutation occurred in each of the following: *APC* (S1545fs), *BRCA2* (V220fs/D2242fs), *CTNNB1* (G34V), *FBXW7* (R465H), *IDH1* (R132S), *PTEN* (Q214X), *SMO* (L412F), *FOXO3* (L382fs), and *PTCH1* (Q694fs) (Fig. 3). TML was lower than 10 per Mb in all of 7 medulloblastomas analyzed (Fig. 3). We did not detect gene amplifications in *EGFR* (n = 8) or *Her2* (n = 16) by FISH and CISH, respectively; MYCN amplification was seen in one tumor using NGS.

## Discussion

This study analysis was based on: (1) therapeutic biomarker expression in the CLIA domain necessary for patient selection and/or stratification for a treatment modality, and (2) the availability of an associated clinical therapeutic. Although molecular subgrouping for medulloblastoma is important for diagnosis and prognosis, this categorization strategy is insufficient for the selection of therapeutics at this time and was not an intent of this study. The most frequent medulloblastoma subgroup in children and second in adults (group 4), and the subgroup with worse prognosis (group 3), lack targeted agents, and prior attempts to match targeted therapeutics with a subgroup have not been therapeutically beneficial [5]. One could argue that a dogmatic requirement of medulloblastoma subtype alignment would be analogous to requiring all “omic” data on glioblastoma be aligned with one of the new molecular subtypes, which have been continuously redefined [13]. Currently, IHC markers for medulloblastoma subtyping, such as WNT, GAB1 or YAP1, are not universally performed and subtyping by IHC does not always coincide with subtyping by other methods (Nanostring, 450k array profiling),[14] indicating that these IHC markers cannot yet be used to select therapeutics for patients. Although our analysis panel included genes previously identified by largescale profiling studies of medulloblastoma, such as CTNNB1 or SMO, it did not include PRDM6 and TERT mutations; however, there are no therapeutics targeted to these alterations at this time, and as such, these have not been included in our focused therapeutic target profiling.

Based on our current study, several identified therapeutic targets have not been previously considered for medulloblastoma such as TS, a marker of cell proliferation and poor prognosis in other solid tumors [15]. Several available TS inhibitors such as raltitrexed, nolatrexed, ZD9331, and OSI-7904L could be considered therapeutically. Perhaps most interesting was expression of the topoisomerase family. We found frequent TOP2A expression, which has been previously noted in medulloblastoma [16]. There are several inhibitors available, such as etoposide, epirubicin, WP744/berubicin and S16020, which could be considered in the context of clinical trials. We also found TOP1 expression, which may have been an unappreciated confounder of response to irinotecan in children with recurrent medulloblastoma [17]. Previously, several clinical trials using irinotecan have reported a signal of response in a subset of patients [17, 18]; however, these trials were conducted before the era of precision medicine, and thus the correlation between treatment response and tumor TOP1 expression was not evaluated. Similar comments can be made for clinical trials of topotecan [19]—another prototypical TOP1 inhibitor. Given frequent TOP1 expression in metastatic tumors and association with TOP2A expression, use of a TOP1 inhibitor in combination with a TOP2A inhibitor may be considered for recurrent and metastatic medulloblastomas. Our analyses also include markers that have been reported to be associated with diminished effectiveness of therapeutic agents. For example, the frequent expression of RRMI and TUBB3 would suggest a lack of benefit to gemcitabine-based chemotherapy [20] and microtubule inhibitors such as epothilones [21], respectively. Conversely, relatively low expression frequency of the excision repair cross-complementation group 1 (ERCC1), which a predictive biomarker of cisplatin-based chemotherapy resistance [22, 23] would indicate current use of cisplatin for medulloblastoma treatment is justifiable in most cases.

Although there is enthusiasm for the use of immune checkpoint inhibitors for treating multiple malignancies, and several studies showed PD-1 and PD-L1 expression in murine models of medulloblastoma [24, 25], the relatively low levels of PD-1-expressing T cells, tumor expressed PD-L1, and tumor mutational burden in medulloblastomas, consistent with a prior report [26], indicate that immune checkpoint inhibitors as a monotherapy should not necessarily be prioritized for therapeutic considerations based on biomarker expression. These findings are also consistent with a prior report of low PD-L1 expression in pediatric cancers [27].

The absence of Her2 or EGFR amplification in our study was not surprising because largescale studies have not identified these cytogenetic abnormalities. MYCN copy number alteration (amplification) was seen in one tumor while CDK6 amplification was not seen. YAP1 amplification, even though identified as characteristic for various subgroups of medulloblastoma was not assessed in this study. The absence of identifiable mutations in many of the samples attest to the potential limitations of targeted strategies for all patients. Furthermore, the rarity of targets found in large data sets indicate that large-scale profiling initiatives would be required in order to identify select subsets of applicable patients. Notably, even in the setting in which a specific targeted agent is used in a selected biomarker-positive population, cellular clonotypic heterogeneity can result in the rapid selection and expansion of non-expressing cells.

As this is a commercial repository for molecular profiling, validated clinical data regarding the treatment courses and patient prognoses is also not available. Hence, we are not able to exclude the possibilities that the expression levels of designated markers, especially in recurrent and metastatic tumors, might have been influenced by therapeutic intervention. Recent studies have revealed that while recurrent and metastatic medulloblastomas retain the same subtype designation as the primary tumor [28, 29], the recurrent and metastatic tumors are genetically distinct from the primary tumor [30, 31]. This cohort includes relapsed and metastatic tumors, which probably influences the molecular profile; however the specimens are not clinically annotated to place biomarker expression in the context of treatment resistance. Notably, our analyzed cohort does reflect the composition of patients that pose a treatment challenge to the clinician. As such, the analysis provides a new perspective for identifying potential therapeutic options outside of the current molecular subtype designations, which may be beneficial for patients. In summary, therapeutics are available for several frequently expressed targets providing a justification for their consideration in future clinical trials for medulloblastoma.

## Electronic supplementary material

Below is the link to the electronic supplementary material.

Supplementary material 1 (DOCX 12 KB)

Supplementary material 2 (DOCX 18 KB)

Supplementary material 3 (DOCX 15 KB)

Supplementary Figure 1: mRNA expression levels in medulloblastoma patients (n=47) from TCGA.
