# Supplementary material for: Rethinking medulloblastoma from a targeted therapeutics perspective
Source: J Neurooncol. 2018 Jun 5;139(3):713–20. doi: 10.1007/s11060-018-2917-2 (PMC6132970; doi:10.1007/s11060-018-2917-2)
Supplement: Supplementary file 1 — Supplementary material 1 (DOCX 12 KB) [file 11060_2018_2917_MOESM1_ESM.docx]

| **Population** | **Primary Tumor Site** | **Specimen Site** | **Recurrent*** |
| --- | --- | --- | --- |
| Pediatric | Cerebellum. NOS | Cerebellum. NOS |  |
|  | Cerebellum. NOS | Cerebellum |  |
|  | Cerebellum. NOS | Cerebellum |  |
|  | Brain. NOS | Brain |  |
|  | Posterior cranial fossa | Posterior cranial fossa |  |
|  | Cerebellum. NOS | Cerebellum. NOS |  |
|  | Cerebellum. NOS | Cerebellum. NOS |  |
|  | Cerebellum. NOS |  |  |
|  | Cerebellum. NOS | Cerebellum |  |
|  | Posterior cranial fossa | Posterior cranial fossa |  |
|  | Posterior cranial fossa | Posterior cranial fossa |  |
|  | Central nervous system | Central nervous system |  |
|  | Brain. NOS | Cerebellum |  |
|  | Cerebellum. NOS | Rib. sternum. clavicle and associated joints |  |
|  | Cerebellum. NOS | Cerebellum |  |
|  | Brain. NOS | Brain. NOS | Yes |
|  | Cerebellum. NOS | Cerebellum. NOS |  |
|  | Cerebellum. NOS | Cerebellum. NOS |  |
| Adult | Temporal lobe | Temporal lobe | Yes |
|  | Parietal lobe | Parietal lobe |  |
|  | Cerebellum. NOS | Cerebellum. NOS | Yes |
|  | Posterior cranial fossa | Posterior cranial fossa | Yes |
|  | Brain. NOS | Dura. NOS |  |
|  | Cerebellum. NOS | Cerebellum | Yes |
|  | Posterior cranial fossa | Cerebellum. NOS |  |
|  | Cerebellum. NOS | Cerebellum. NOS |  |
|  | Brain. NOS | Ventricle |  |
|  | Cerebellum. NOS | Brain. NOS | Yes |
|  | Brain. NOS | Brain. NOS |  |
|  | Cerebellum. NOS | Cerebellum. NOS | Yes |
|  | Brain. NOS | Cerebellum | Yes |
|  | Cerebellum. NOS | Cerebellum |  |
|  | Brain. NOS | Brain |  |
|  | Brain. NOS | Brain. & Cranial Nerves. & Spinal Cord. (Excl. Ventricle. Cerebellum) |  |
|  | Spinal cord | Spinal cord | Yes |
|  | Brain. NOS | Lymph nodes |  |

**Supplementary Table 1: Anatomical location of the specimens**

*Designated by the referring physician
