# Supplementary material for: Rethinking medulloblastoma from a targeted therapeutics perspective
Source: J Neurooncol. 2018 Jun 5;139(3):713–20. doi: 10.1007/s11060-018-2917-2 (PMC6132970; doi:10.1007/s11060-018-2917-2)
Supplement: Supplementary file 2 — Supplementary material 2 (DOCX 18 KB) [file 11060_2018_2917_MOESM2_ESM.docx]

|  |  | **Pediatric** | **Adult** |
| --- | --- | --- | --- |
| **CISH** | | 8 | 8 |
| **FISH** |  | 4 | 4 |
| **IHC** |  | 18 | 18 |
| **NextGen SEQ** | | 9 | 10 |
|  | NextSeq | 4 | 3 |
|  | Non-posterior fossa, *n* (%) | 5 | 7 |

**Supplementary Table 2: Number of medulloblastoma cases tested with different technologies**
