# Supplementary material for: Rethinking medulloblastoma from a targeted therapeutics perspective
Source: J Neurooncol. 2018 Jun 5;139(3):713–20. doi: 10.1007/s11060-018-2917-2 (PMC6132970; doi:10.1007/s11060-018-2917-2)
Supplement: Supplementary file 3 — Supplementary material 3 (DOCX 15 KB) [file 11060_2018_2917_MOESM3_ESM.docx]

| **Protein** | **Negative Threshold** | **Positive Threshold** | **Primary Antibody** |
| --- | --- | --- | --- |
| AR | 0 or <10% | ≥1+ and ≥10% | AR27 |
| PR | 0 or <10% | ≥1+ and ≥10% | 1E2/100 |
| ER | 0 or <10% | ≥1+ and ≥10% | SP1 |
| Her2 | ≤1+ or =2+ and ≤10% | ≥3+ and >10% | 4B5 |
| EGFR | 0 or <10% | ≥1+ and ≥10% | H11 |
| cMET | <50% or <2+ | ≥2+ and ≥50% | SP44 |
| TOPO1 | 0 or <30% or <2+ | ≥2+ and ≥30% | 1D6 |
| TOPO2A | 0 or <10% | ≥1+ and ≥10% | 3F6 |
| TS | 0 or ≤3+ and <10% | ≥1+ and ≥10% | TS106/4H4B1 |
| TUBB3 | <2+ or <30% | ≥2+ and ≥30% | Polyclonal |
| PGP | 0 or <10% | ≥1+ and ≥10% | C494 |
| MGMT | 0 or ≤35% | ≥1+ and >35% | MT23.3 |
| PTEN | 0 or ≤50% | ≥1+ and >50% | 6H2.1 |
| TLE3 | <30% or <2+ | ≥2+ and ≥30% | Polyclonal |
| RRM1 | 0 or <50%  or <2+ | or ≥2+ and ≥50% | Polyclonal |
| ERCC1 | <2+ or ≤3+ and <10% or =2+ and <50% | ≥3+ and ≥10% or ≥2+ and ≥50% | 8F1 |
| PD-1 | 0 | ≥1+ | MRQ-22 |
| PD-L1 | <2+ or ≥2+ or <5% (tumor staining) | ≥5% (tumor staining) | 130021/SP142 |
| SPARC m/p | <2+ or <30% | ≥2+ and ≥30% | 12251 |

**Supplementary Table 3: Immunohistochemistry antibodies and thresholds for categorization**

***** IHCs are read as: intensity (0, 1+, 2+, 3+) and percentage staining

**REFERENCES:**

Gucalp A, Tolaney S, Isakoff SJ, et al. Phase II trial of bicalutamide in patients with androgen receptor-positive, estrogen receptor-negative metastatic Breast Cancer. Clin Cancer Res. 2013 Oct 1;19(19):5505-12.

Lewis JD, Chagpar AB, Shaughnessy EA, et al. Excellent outcomes with adjuvant toremifene or tamoxifen in early stage breast cancer. *Cancer*. 2010;116(10):2307-2315

Bartley AN, Washington MK, Colasacco C et al. HER2 Testing and Clinical Decision Making in Gastroesophageal Adenocarcinoma: Guideline From the College of American Pathologists, American Society for Clinical Pathology, and the American Society of Clinical Oncology. J Clin Oncol. 2017 Feb;35(4):446-464.

Park K, Han S, Shin E, et al. EGFR gene and protein expression in breast cancers. *Eur J Surg Oncol*. 2007;33(8):956-960

Dziadziuszko R, Wynes MW, Singh S, et al. Correlation between MET gene copy number by silver in situ hybridization and protein expression by immunohistochemistry in non-small cell lung cancer. *J Thorac Oncol*. 2012;7(2):340-347.

Braun MS, Richman SD, Quirke P, et al. Predictive biomarkers of chemotherapy efficacy in colorectal cancer: results from the UK MRC FOCUS trial. *J Clin Oncol.* 2008;26(16):2690-2698.

Durbecq V, Paesmans M, Cardoso F, et al. Topoisomerase-II alpha expression as a predictive marker in a population of advanced breast cancer patients randomly treated either with single-agent doxorubicin or single-agent docetaxel. *Mol Cancer Ther.* 2004;3(10):1207-1214.

Sun JM, Ahn JS, Jung SH, et al. Pemetrexed Plus Cisplatin Versus Gemcitabine Plus Cisplatin According to Thymidylate Synthase Expression in Nonsquamous Non-Small-Cell Lung Cancer: A Biomarker-Stratified Randomized Phase II Trial. *J Clin Oncol.* 2015;33(22):2450-2456.

Yeh JJ, Hsu WH, Wang JJ, et al. Predicting chemotherapy response to paclitaxel-based therapy in advanced non-small-cell lung cancer with P-glycoprotein expression. *Respiration*. 2003;70(1):32-35.

Sève P, Isaac S, Trédan O, et al. Expression of class III {beta}-tubulin is predictive of patient outcome in patients with non-small cell lung cancer receiving vinorelbine-based chemotherapy*. Clin Cancer Res.* 2005;11(15):5481-5486.

Ma S, Egyházi S, Ueno T, et al. O6-methylguanine-DNA-methyltransferase expression and gene polymorphisms in relation to chemotherapeutic response in metastatic melanoma. *Br J Cancer*. 2003 89(8):1517-1523.

Razis E, Bobos M, Kotoula V, et al. Evaluation of the association of PIK3CA mutations and PTEN loss with efficacy of trastuzumab therapy in metastatic breast cancer. *Breast Cancer Res Treat.* 2011;128(2):447-456

Kulkarni SA, Hicks DG, Watroba NL, et al. TLE3 as a candidate biomarker of response to taxane therapy. *Breast Cancer Res*. 2009;11(2):R17.

Maréchal R, Bachet JB, Mackey JR, et al., Levels of gemcitabine transport and metabolism proteins predict survival times of patients treated with gemcitabine for pancreatic adenocarcinoma. *Gastroenterology*. 2012;143(3):664-674.e6

Scheil-Bertram S, Tylus-Schaaf P, du Bois A, et al., Excision repair cross-complementation group 1 protein overexpression as a predictor of poor survival for high-grade serous ovarian adenocarcinoma. *Gynecol Oncol.* 2010; 119(2):325-331

Ghebeh H, Mohammed S, Al-Omair A, et al., The B7-H1 (PD-L1) T lymphocyte-inhibitory molecule is expressed in breast cancer patients with infiltrating ductal carcinoma: correlation with important high-risk prognostic factors. *Neoplasia*. 2006;8(3):190-198.

Robert C, Long GV, Brady B, et al. Nivolumab in previously untreated melanoma without BRAF mutation. *N Engl J Med*. 2015;372(4):320-330

Desai N, Trieu V, Damascelli B, et al., SPARC Expression Correlates with Tumor Response to Albumin-Bound Paclitaxel in Head and Neck Cancer Patients. *Transl Oncol.* 2009;2(2):59-64.
