# Supplementary figures and images for: Rethinking medulloblastoma from a targeted therapeutics perspective
Source: J Neurooncol. 2018 Jun 5;139(3):713–20. doi: 10.1007/s11060-018-2917-2 (PMC6132970; doi:10.1007/s11060-018-2917-2)

## Slide 1
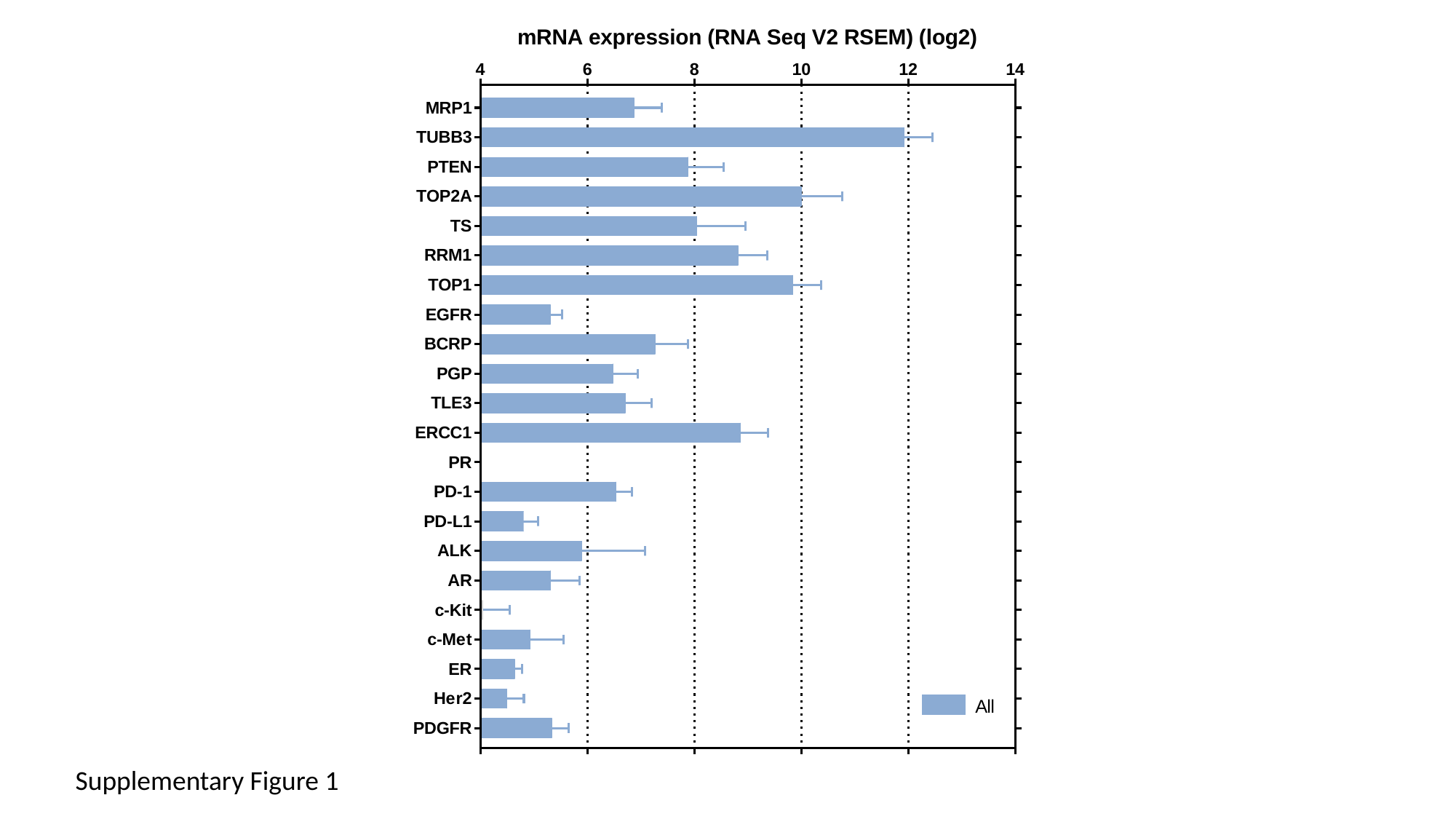

Supplementary Figure 1

Supplement: Supplementary file 4 — Supplementary Figure 1: mRNA expression levels in medulloblastoma patients (n=47) from TCGA. [file 11060_2018_2917_MOESM4_ESM.pptx]
